# Caring for a stroke patient: The burden and experiences of primary caregivers in Uganda – A qualitative study

**DOI:** 10.1002/nop2.356

**Published:** 2019-08-04

**Authors:** Namale Gertrude, Rachel Kawuma, Winifred Nalukenge, Onesmus Kamacooko, Laetitia Yperzeele, Patrick Cras, Edward Ddumba, Robert Newton, Janet Seeley

**Affiliations:** ^1^ MRC/UVRI and LSHTM Uganda Research Unit Entebbe Uganda; ^2^ Department of Neurology, Faculty of Medicine and Health Sciences, Born Bunge Institute, University of Antwerp Antwerp University Hospital Antwerp Belgium; ^3^ St. Francis Hospital Nsambya affiliated to Uganda Martyrs University Kampala Uganda; ^4^ University of York York UK; ^5^ London School of Hygiene &Tropical Medicine London UK

**Keywords:** burden, experiences, nurses, nursing, patient, primary caregiver, stroke, Uganda

## Abstract

**Aim:**

We assessed the burden and experiences of caregivers looking after stroke patients in Kampala, Uganda.

**Design:**

We conducted a qualitative cross‐sectional study between May 2018–July 2018 among primary caregivers of stroke patients.

**Methods:**

The primary caregiver was defined as the person spending most of the time providing daily care for the stroke patient for at least four months. Purposive sampling was used to consecutively recruit the primary caregivers. In‐depth interviews were conducted, and audiotape recorded, and observations were also made. Data were managed using NVIVO 12.0 following thematic approach.

**Results:**

Twenty‐five caregivers were included in the analysis with a mean age of 39.3, *SD* 10.7. Four themes were identified from the qualitative analysis on caregivers’ experiences of looking after stroke patients: taking on new responsibilities, factors that protected caregivers from breaking down, limited resources and experiences with patient outcomes. Our findings highlight the need for interventions to support stroke patients and their caregivers.

## 1 INTRODUCTION

Stroke remains one of the most devastating of all the non‐communicable and neurological diseases, often causing death or gross physical impairment and disability (Strong, Mathers, & Bonita, 2007). Globally, two‐thirds of stroke survivors return home in spite of their disabilities (Strong et al., 2007). More than 85% of all stroke deaths and disability occur in low‐ and middle‐income countries, including sub‐Saharan Africa (SSA) (Connor, Walker, Modi, & Warlow, 2007). In Uganda, it accounts for one of the top 10 causes of hospitalization and is a major cause of chronic disease (Guwatudde et al., 2015). The responsibility for care after discharge is often transferred to a family member with no previous experience of caring for someone who has had a stroke.

### 1.1 Background

Primary caregivers have an important role in caring for stroke patients (Ae‐Ngibise, Doku, Asante, & Owusu‐Agyei, 2015; Greenwood, Mackenzie, Cloud, & Wilson, 2009; Igberase, Morakinyo, Lawani, James, & Omoaregba, 2012). The needs of a stroke survivor may vary from being physical (help walking, carrying from bed to toilet), in communication and nursing (feeding, personal hygiene) (Ae‐Ngibise et al., 2015). These primary caregivers must not only learn to meet the needs of the stroke survivor, but also cope with their own fears and anxieties as they are suddenly cut‐off from their old lifestyle and thrown into a new role (Masuku, Mophosho, & Tshabalala, 2018). Women bear the greatest burden of caregiving, with 59%–75% of caregivers being women (Alliance, 2003). The reduced employment commitments related to caregiving or leaving paid work to provide unpaid care leads to financial losses, reduced family income and financial difficulties (Saban & Hogan, 2012; Wagachchige Muthucumarana, Samarasinghe, & Elgán, 2018).

Recent research suggests that caregivers who subjectively reported a high amount of burden also reported poorer physical functioning and more emotional distress than other caregivers (Igberase et al., 2012). Caregiver burden has been estimated to be between 20%–40% (Sawatzky & Fowler‐Kerry, 2003). Qualitative studies described caregivers’ reactions after the stroke occurred, including feelings of confusion and lack of preparation for the caring role (Greenwood et al., 2009). Previous studies have also identified, the frequent difficulties described by caregivers including physical, social and emotional, more of the household chores and financial aspects (Ae‐Ngibise et al., 2015). Some caregivers have emphasized the need for information, training and support to help them gain greater confidence in the care they provide (Strong et al., 2007).

For SSA including Uganda, research on stroke caregivers is scarce. In this paper, we contribute to that research by documenting the burden and experiences of caregivers looking after stroke patients in Uganda.

### 1.2 Design

A descriptive cross‐sectional study was conducted from May 2018–July 2018 as part of an epidemiological stroke study conducted at Nsambya hospital, a private hospital located in a peri‐urban suburb in south‐eastern Kampala, the capital city of Uganda.

## 2 METHODS

### 2.1 Sample selection, participants and recruitment

A sample of 25 primary caregivers was consecutively recruited for this study. Purposive sampling was used to recruit as varied a sample as possible to gather a wide range of responses. The interviewer compiled a list of potential participants, and the selection of each participant was based on choosing a range of people representing participants of different age, gender, educational level, duration of caring experience and geographical location. Purposive sampling was used to ensure maximum variation of the sample and caregiver experiences. The participants were self‐reported primary caregivers that took care of stroke patients most of the time. They were subsequently confirmed by review of medical records. The primary caregiver was defined as the person spending most time providing daily care for the stroke patient or the person taking on the main caregiving roles for at least four months. Potential participants were identified using the register compiled as part of the stroke patient list of the main stroke study. Caregivers of stroke inpatients were approached by the study nurse or the research assistant, given information and invited to participate. Caretakers in the community were recruited using telephone contacts, and those who responded positively were invited to participate in the interviews at Nsambya hospital.

### 2.2 Eligibility

Participants were eligible if they were aged 18 years old or older, able to speak English or Luganda and giving care to a stroke patient at home or in hospital for at least four months, being the primary caregiver and be willing to sign a consent form. Families where the main caregiver could not be identified were excluded.

### 2.3 Data collection procedure

Face‐to‐face in‐depth interviews were conducted by the first author and an experienced social scientist in the caregivers’ preferred language (English or Luganda). The interviews lasted between lasted 45–60 min and were audiotaped with participants’ consent. We used an open‐ended interview guide which was revised through two pilot interviews. Data collection took place at a convenient time for the caregivers in a quiet place after obtaining written‐informed consent. All data were collected either in the caregivers’ homes or in a private office at Nsambya hospital, depending on the preference of the caregiver. Interviews were conducted after patients were discharged or just prior to discharge. The participants were asked to describe their experiences and feelings in relation to their caregiving duties. The interviews were conducted in private to enable the caregiver to respond freely. Interviews were transcribed and translated into English.

### 2.4 Data analysis and quality assessment

For qualitative data, all transcribed verbatim and interview notes were analysed after critical and frequent reading of the transcripts. A coding framework was developed based on priori themes used in the study design and themes which emerged from the data. NVivo 12.0 qualitative software was then used to code the transcripts. The coding was discussed and verified by investigators. The data from each participant were coded by the first author and discussed with the second, third and last author. We used the STrengthening the Reporting of OBservational studies in Epidemiology (STROBE) cross‐sectional reporting guidelines for methodological quality assessment (Von Elm et al., 2014).

### 2.5 Ethical considerations

Research Ethics Committee approval to conduct this study was obtained from the Uganda Virus Research Institute Research and Ethics Committee and the Uganda National Council for Science and Technology. Written‐informed consent was obtained from the research participants before performing any study procedures. To maintain participant confidentiality, all study data were collected using only numerical unique identifiers. All caregivers signed and remained with a copy of the consent form before participation in the study.

### 2.6 Data sharing

The data collected in this study is suitable for sharing and procedures for accessing it is contained in the data‐sharing policy accessible from the Medical Research Council (MRC) website (https://www.mrcuganda.org/publications/data-sharing-policy).

## 3 RESULTS

### 3.1 Recruitment profile

During the study period, 38 eligible caregivers were purposively selected to participate in the study. Of these, three could not be contacted as per the contact details provided during enrolment. Of the 35 who were contacted, seven were unwilling to participate (three were still grieving and were too emotional to attend the session, three were nursing very ill stroke patients and there was no other helper at home, one had a very busy schedule at work) and three could not make it to the interview due to long distances and high transport costs. Thus, a total of 25 caregivers were included in the analysis.

### 3.2 Socio‐demographic characteristics

Of the 25 caregivers were included in the analysis, most (*N* = 14) of them were females. The mean age was 39.3, ranging from 22–66 years. More than half (*N* = 15) were of 35–54 years, eight were of 18–34 years, and only two were ≥ 55 years. Nine caregivers were children of the stroke patients, nine were spouses, while the seven were either siblings or friends. Most (*n* = 20) attained secondary and tertiary education and more than half were married/cohabiting (*n* = 17). Nine were in formal employment and self‐employed, and only one participant was a student. The duration of caregiving varied from four months–12 years. The median duration of caregiving was 16 months. At the time of interview, 21 stroke patients were still alive and the frequent stroke type among the stroke patients was ischaemic stroke (*N* = 15) (Table 1).

**Table 1:** Characteristics of caregivers of stroke patients in Kampala, Uganda

Caregiver and patient characteristics	*N* = 25 *n* (col %)
Gender	
Male	11 (44)
Female	14 (56)
Age	
18–34	8 (32)
35–54	15 (60)
>=55	2 (8)
Relationship with stroke patient	
Spouse	9 (36)
Child/Child in law	9 (36)
Other	7 (28)
Marital status	
Single/never married	4 (16)
Married/Cohabiting	17 (68)
Widowed	1 (4)
Separated/Divorced	3 (12)
Duration of care	
<1 year	10 (40)
>=1 year	15 (60)
Education level	
Primary	5 (20)
Secondary	10 (40)
Tertiary	10 (40)
Occupation	
Formal employment	9 (36)
Farmer	3 (12)
Self‐employed	9 (36)
Unemployed	3 (12)
Student	1 (4)
Type of stroke	
Ischaemic stroke	15 (60)
Haemorrhagic stroke	8 (32)
Missing	2 (8)

Four themes were identified from the qualitative analysis on caregivers’ experiences and burden of looking after stroke patients: taking on new responsibilities, factors that protected caregivers from breaking down, limited resources and experiences with patient outcomes.

### 3.3 Theme 1: Experiences of taking on new responsibilities

Caregivers reported that they were taking on new responsibilities and began to feel the caregiver demands placed on them. Their lifestyles, routines and life status were significantly altered. The stroke was an abrupt and unexpected event and caregivers found themselves in a new life situation where they suddenly had to take on new responsibilities of caring for their relative:

I’m affected financially for there are many people, around 10 of them in the home and I have to care for everybody. You look for their food, clothing, school fees, so you have to make an adequate budget for all that, while you have to get medication for the patient. (41‐year‐old female sibling)

Caregivers described an increased workload from their new caregiving roles. Such demands included assisting the stroke patient in their daily activities, such as assisting them to go to the toilet, dressing, feeding, dispensing drugs, as well as providing emotional and psychological support. Some caregivers reported difficulty finding a balance between their job, their family and caregiving responsibilities. Some relatives complained of body pain and fatigue:

I was working in some research company, but I couldn’t cope with it because I had to take care of her. I got some health problem. My legs started swelling because we would sit the whole night that is if my mum sleeps, then me I would sit. And we would sleep on the floor. Yet we had to feed her at intervals. You had to keep awake many times as if you were waiting for something. Yeah sleepless nights and mornings. (32 ‐year ‐old granddaughter)

The caregivers suddenly had to learn new skills to cope with their stroke patient's new situation. They said that they had a lot more to learn about caring for a stroke patient, for example, the skills on turning the patient, feeding, exercises and dispensing medications:

I used to tell her to get hold on my shoulders for support, I would make her walk a bit in the morning before having her breakfast, because a certain medical staff had told me that, to make her do exercises… (56 ‐year ‐old daughter)

I used to carry her, I used to shower her, I used to feed her through the tubes because the nurse had told us to do it, … sometimes I would go and ask the nurse how to give the medicine…, I would get those green vegetables from our garden at home, prepare them for her, since they told us they are good, then also massage her sometimes, the doctor had told us to massage her a little bit. (22 ‐year ‐old granddaughter)

### 3.4 Theme 2: Factors that protected caregivers from breaking down

While caregivers had to cope with a new and challenging situation, this experience for some also had positive effects because of the emotional attachment and affection they had towards their patients. To a 39‐year‐old female sibling it was; “I used to do everything out of love; leave alone the money for I was focused on improving his health”, while a 40 year ‐old husband said “we vowed to be together during time of happiness and time sorrows; I can't be away”.

For many caregivers, their spiritual beliefs played a positive and effective role in maintaining their hope, to keep them moving and remain strong. These caregivers tried their best to enhance their spiritual aspects by doing several activities as a 39‐year‐old wife said: “I usually read the bible messages, so as to be positive and to remain strong”. A 40‐year‐old husband said; ‘recently we went to the pastor in the church, who came and prayed for us, encouraged us, read for us bible verses which brought us hope…’ However, some caregivers consulted traditional healers, rather than religious leaders, as a 39‐year‐old female sibling narrates:

….for us we went to a certain village as we were told that she was suffering from a traditional illness and we went to the traditional healer who charged us so much and he is still demanding for his money for treating the patient. The traditional healer said that, this illness was related to the clan family spirits; they were demanding for her life.

Supportive social networks of the family members, relatives, friends and neighbours were important in helping caregivers cope with the increasing caregiving demand:

Our neighbours and family have also given us support like one neighbour gave us her daughter to take care of our home and she looks after the children. She is the one who keeps the home tidy and clean, she cooks. While also this patient’s friends keep on giving us some money. …. and buy food and put it at home to sustain the children. (41‐year‐old male sibling)

Caregivers appreciated support from the healthcare providers who really listened to their concerns and they spoke positively about them. Such support enabled caregivers cope in their new roles and provided them with the confidence they needed:

The hospital staff were good because we would come and that doctor was good. She could come and counsel us even when we were admitted, it was a good life here. We were given a good reception. (a 48‐year‐old wife)

### 3.5 Theme 3: Limited resources in care of stroke patients

The caregivers frequently mentioned that the increase in demands was difficult because of the limited resources such as food, gloves, face masks and wheelchairs:

I wanted support like to feed the patient with fruit, for they used to be scarce. Like I have told you, I stopped working otherwise I would have bought fruit, milk for she used to like drinking more that eating, oil for messaging the affected side of the body, which was so expensive at 16,000/‐ (approximately £4). (22 ‐year ‐old granddaughter)

….there was a time when the patient was coughing and the wounds were so smelly. The hospital would have given us gloves and powder to take care of those wounds at home, even those face masks to put on and yet we did not have money to buy them. (32‐ year‐ old granddaughter)

The loss of income of either the stroke patient or of the caregiver was a major reason for financial problems. For example, a 39‐year‐old woman caring for her husband was not able to afford a physiotherapist and wheelchair to help his wife. Some were even forced to borrow money to take care of their stroke patients.

As a result, some caregivers gave up treatment for their stroke patients, as they could no longer sustain such big costs:

The main challenge that I faced was failure to bring her for the check‐up; because even before she died she used to urge us to take her for this review; but we could not afford it. (22‐year‐old granddaughter)

Other caregivers wished to be visited and their patients be reviewed from home to cut on the costs. A 48‐year‐old husband said that “I wish there is a process of visiting stroke patients in their homes, some nurses to go door to door to watch over these stroke patients because it is not easy, it's expensive”. But some wished that the government would offer support to caregivers and stroke patients for example drugs and items for caring (such as gloves).

Whereas some caregivers expressed lack of resources such as finances and food, other caregivers expressed inadequate support from the healthcare providers:

It is such a hard situation when the attendant runs to a medical staff for attention, instead of being prompt and cooperative, such staff don’t care at all! Otherwise the medical staff feel disgusted about the stroke patients. Because most of the patients come when they are very sick. There are very few nurses that give such attention to such stroke patients. (45‐year‐old male sibling)

Similarly, a 48‐year‐old husband said:

Maybe you should also find a way how you are going to handle the issue of medical staff that meet the patients and now it looks as if it is to whom it may concern […]You look at the medical staff who has put on something as a medical uniform and she asks you: who have you come with! a medical staff would ask such a question! Go for the scan and come back! You must bring her back! ….and I was alone with my patient! You move! Go up there! Go to that end! Then I said, what was all that! It stresses patients by the way, it stresses a lot.

### 3.6 Theme 4: Experiences with patient outcomes

Some caregivers reported that their stroke patients had recovered. They felt happy and relieved getting their family members back at home. This relief was also related to having less involvement in caregiving than they had experienced before. This brought them great pleasure as a 32‐year‐old male sibling described this as follows:

…now she can sit up right, that is a great pleasure and a relief to me. She can now breastfeed her baby, when I support her like this, she can be able to move using the strong leg.

However, when patients died, there was psychological distress and grief following loss of their spouses, relatives and friends due to stroke. This deeply affected many aspects of their lives in different ways as narrated below:

Yes, the hardest moment was when I was calling mum and she couldn’t open her mouth, she was gone… that was the hardest moment […] and the situation has really not been easy. It has affected me so much as an individual. Not me alone but also my siblings. I lost my job because I needed to take care of mum […]. now I am struggling to survive, everything is hard now, yet they have never been hard. All that has happened after mum died. (22‐ year‐ old daughter)

## 4 DISCUSSION

In this study, we present findings which highlight the unique experiences of caring for a stroke patient in a home setting. The four broad thematic areas identified in this study were similar to those from previous studies (Ae‐Ngibise et al., 2015; Masuku et al., 2018; Simeone et al., 2016). Overall, the process of caring described by the caregivers in this study illustrates challenging experiences and a difficult caring journey.

The high number of women caregivers in the current study is similar to previous literature (Ae‐Ngibise et al., 2015; Salama, 2012). This finding reflects the Ugandan culture, where caregiving is viewed as a woman's role even if she has fulltime employment. The women are commonly devoted to caring for their family members and for managing household chores. On the other hand, the male caregivers were also undertaking the challenge of caring for their stroke patients. Most male caregivers were providing care by themselves and did not depend on other people. A previous study in Japan (Uemura, Sekido, & Tanioka, 2014) showed that male caregivers learned how to provide care by themselves without support.

The significant life changes which occurred in the caregivers’ lives were mainly due to the sudden assumption of new responsibilities, increased workload, changes in domestic chores, sadness and changed expectations of life. The changes in the stroke caregivers’ lives are a recurrent finding in the literature (Ang et al., 2013; Bulley, Shiels, Wilkie, & Salisbury, 2010). Previous reports have indicated that caregiver life changes may differ depending on the caregiver relationship with the stroke patient (Ang et al., 2013). The spouses in this study experienced more anxiety than children or siblings and unrelated caregivers experienced the least anxiety. However, since these caregivers were mostly women, the sudden life changes may have important aspects about balancing caregiving with other demands including child‐bearing, career, relationships and household chores.

These constrained lives with increased workload at home and caring responsibilities led to feeling overwhelmed. The life at home had to be juggled alongside the stroke crisis and the demands of looking for school fees, feeding, treatment and transport to the health facility. Consistent with previous reports, the high levels of stress and burden of caregiving made it difficult to find balance between caregiving, family, job and personal life (Simeone et al., 2016). These roles were so demanding and stressful often described as physical fatigue, poor sleep and body pain. The tiredness and exhaustion reported by our caregivers have been documented in other studies as well (Bulley et al., 2010; Masuku et al., 2018; Wagachchige Muthucumarana et al., 2018)***.*** In contrast, because of the available social support systems, caregivers in Japan were less stressed and had a lower sense of care burden (Uemura et al., 2014). Probably, because there are currently no rehabilitation support services in Ugandan communities, this could have had an impact on the continuous stress in these caregivers. This finding provides interesting opportunities for intervention by healthcare professionals. There is need for strong institutional and professional support for caregivers of stroke patients in the country to reduce their caregiver burden.

Many caregivers in this study were employed and had families. In fact, ten caregivers had to lose their jobs because of caregiving responsibilities. This led to financial constraints, needing some support from friends, family and government institutions. For example, some caregivers wished if the government could offer them free stroke medicines and wheelchairs. For the underprivileged families, to reduce the burden of costs, caregivers sometimes provided herbal medicine to their patients and some even stopped coming back for hospital reviews. Caregivers in South Africa (Mashau, Netshandama, & Mudau, 2016), experienced similar challenges, such as shortages of nursing supplies and could not always protect themselves against exposure to infections. However, one would ask a question why these caregivers did not retain their jobs and find an unemployed family member or friend to perform their caregiving duties. Probably the sense of love, spiritual fulfilment, a sense of duty and social pressures which were frequently highlighted are some of the reasons why these caregivers had to keep moving on with their duties.

The critical challenges experienced by these caregivers are similar to most developing countries [6, 11] and a common finding in literature (Ae‐Ngibise et al., 2015; King, Ainsworth, Ronen, & Hartke, 2010; Masuku et al., 2018). The need for information support, skills and active listening to caregivers’ concerns to feel confident about caregiving was very crucial. This is consistent with the findings of a systematic review (Greenwood et al., 2009) which revealed the educational needs of caregivers. It is possible that sometimes healthcare professionals give information only after a caregiver has asked for it. However, the help they received from the family and friends was very well appreciated by these caregivers, as other investigators have also found (Cecil et al., 2011). Because these caregivers experienced a lot of difficulties in care, the family and friends were an important source of support. These family relationships and support reflect the Ugandan traditional extended family function. In our society and culture, the joint family support system helps in dividing the burden and supporting relatives together to improve the patient's health care. This observation is also similar to findings reported in other studies in Ghana (Ae‐Ngibise et al., 2015) and South Africa (Mashau et al., 2016).

Consistent with previous reports, the relief after the patient's recovery is definitely an aspect of positive caregiving (Pierce, Steiner, Govoni, Thompson, & Friedemann, 2007). The perceived benefit in the recovery of the patient allowed these caregivers to devote less time to providing care. Literature has reported that even a slight physical improvement in stroke patients makes caregivers feel relieved (Simeone, Savini, Cohen, Alvaro, & Vellone, 2015). Consistent with previous studies, caregivers who had lost their loved ones due to stroke experienced a lot of psychological distress (Saban & Hogan, 2012). For many people, grief is one of the hardest things that they will have to go through. Importantly, it can be helpful for the bereaved caregivers to speak to a qualified professional about their feelings for appropriate counselling.

Our findings address issues that could be translated into policy and practice. These caregivers have specific needs that could be addressed by Ministry of Health and healthcare professionals. Caregivers offered several suggestions to healthcare professionals and government institutions to improve care of their stroke patients. First, caregivers advised for active listening to their concerns. A flexible and concerned attitude among health professionals which can have a positive effect on caregivers is critical in care and has been documented (Begum, 2014; Bugge, Alexander, & Hagen, 1999; Lee, Yoon, & Kropf, 2007). Secondly, caregivers highlighted the need for training in the basic skills of handling a stroke patient, simple nursing tasks, information support and postdischarge follow‐up which can reduce the burden of care. Thirdly, the caregivers expressed the need for government to extend support to them and their stroke patients with medicines, gloves and wheelchairs especially those who are disadvantaged in communities. Combining these multiple concerns and needs suggested by several caregivers, the authors emphasize that it is very important to support these caregivers as they care for their loved ones either at home or at health facilities to improve the health outcomes for both the stroke patient and caregiver. All these areas can be attended to by the relevant health professionals and policy makers. Caregiver needs could be included as part of a comprehensive approach to stroke care policy in the country.

Our study has some limitations. Generalizability of the findings may be limited due to the use of a purposive sample and also because this is a single‐centre study, the caregivers were recruited from one area, Kampala district. The participants may not be representative of stroke caregivers in the general population.

In summary, caregivers experienced life changes due to sudden assumption of new responsibilities. These caregivers experienced caregiving burden mainly due to financial difficulties, emotional distress, high caregiving demands and a lack of rehabilitation support services in their communities. With the absence of community rehabilitation support services, our caregivers experienced continuous stress in their caregiving roles which had a profound impact on their lives. Many caregivers experienced a lack information support and skills and active listening to their concerns which were crucial during their caregiving duties. The caregivers also experienced difficulties in balancing caregiving, family and personal life and needed support.

We suggest that further research be carried out to ascertain the feasibility of a homecare model in SSA including Ugandan settings, with greater emphasis on the cost‐effectiveness and sustainability of such an intervention.

## CONFLICT OF INTERESTS

The authors declare that they have no competing interests.
